# Longitudinal relationship between baseline Weight-Adjusted Waist Index and stroke risk over 8 years in Chinese adults aged 45 and older: a prospective cohort study

**DOI:** 10.3389/fpubh.2025.1505364

**Published:** 2025-02-12

**Authors:** Xiaoqiang Li, Xiangmao Zhou, Hui Du, Hui Wang, Zhijian Tan, Yaqing Zeng, Zhibin Song, Guifeng Zhang

**Affiliations:** ^1^Department of Neurology, Xiaolan People’s Hospital of Zhongshan (The Fifth People’s Hospital of ZhongShan), Guangdong, China; ^2^Department of Gastrointestinal Surgery, The Central Hospital of Yongzhou, Yongzhou, Hunan, China; ^3^Department of Blood Transfusion, Xiaolan People’s Hospital of Zhongshan (The Fifth People’s Hospital of ZhongShan), Guangdong, China

**Keywords:** Weight-Adjusted Waist Index, obesity, stroke, CHARLS, China adults

## Abstract

**Background:**

This study explores the longitudinal relationship between the Weight-Adjusted Waist Index (WWI), an innovative obesity metric, and stroke incidence in aged 45 and older Chinese adults.

**Methods:**

Data from 9,725 individuals aged 45 years and older were analyzed from the China Health and Retirement Longitudinal Study (2011–2020). Baseline characteristics were analyzed across different quartiles of the WWI. Stroke incidents were determined through self-reported doctor diagnoses. Multivariate logistic regression analyses and curve fitting assessed the WWI-stroke risk relationship, adjusting for various demographic, lifestyle, and health-related factors.

**Results:**

Higher WWI quartiles were associated with older age and higher prevalence of stroke and adverse health conditions. In the fully adjusted model, participants in the highest WWI quartile had an odds ratio of 1.52 (95% CI: 1.19, 1.92) for stroke compared to the lowest quartile. Curve fitting revealed a linear relationship between WWI and stroke risk, consistent across various demographic and clinical subgroups.

**Conclusion:**

Higher WWI is linked to an increased risk of stroke in aged 45 and older Chinese adults over an eight-year period. WWI may serve as an effective tool for predicting long-term stroke risk within this population. However, the study is limited by the reliance on self-reported stroke diagnoses and the presence of potential residual confounding factors.

## Introduction

Stroke, a neurological disorder marked by the sudden loss of brain function due to disrupted blood flow, remains a significant global health concern (1, 2). Epidemiological research have consistently identified stroke as a primary cause of mortality and long-term disability, impacting millions each year (3). In China, stroke is the leading cause of death, with an estimated 17.8 million prevalent cases and 3.4 million new incidents annually as of 2020 (4). The overall age-standardized prevalence and incidence rates in adults aged 40 years and older were 2.6% and 505.2 per 100,000 person-years, respectively (5, 6). Stroke etiology is multifaceted, with numerous factors influencing its occurrence. Key contributors include hypertension, cardiovascular conditions, and diabetes mellitus, which are strongly linked to stroke events (1, 7, 8). Additionally, age-related vascular risk factors are critical in stroke development, particularly in middle-aged and older adults, as their cumulative effects increase over time (9). Among older adult individuals, the risk becomes even more pronounced due to complications such as sarcopenia and inadequate nutritional intake, which can adversely affect stroke prognosis (10). Recent evidence suggests that these aging-related factors significantly influence stroke susceptibility, emphasizing the necessity of thorough risk evaluations for this high-risk group (11). The grave impact of stroke highlights the importance of implementing early detection measures and effective monitoring systems.

Globally, excess weight conditions have reached pandemic proportions, representing a significant challenge to healthcare systems (12, 13). In China, the prevalence of overweight and obesity has increased dramatically over the past decades. Data from 2015 to 2019 reveal that 34.3% of Chinese adults are overweight, with 16.4% classified as obese (14, 15). While Body Mass Index (BMI) and waist circumference (WC) are widely used to evaluate obesity, these traditional measures have significant drawbacks. BMI does not distinguish between fat and muscle mass (16, 17), and WC is strongly correlated with BMI, making it less effective in isolating adiposity-related health risks (18). Although the waist-to-height ratio (WHtR) has been suggested as a potentially superior measure, its ability to predict cardiovascular disease (CVD) and mortality linked to obesity remains debated (19, 20). To address these shortcomings, the WWI has been developed as a novel obesity indicator. By standardizing WC against body weight, WWI retains the advantages of WC while reducing its overlap with BMI, allowing for a clearer distinction between fat and muscle mass (21). Studies, particularly those involving East Asian populations, indicate that WWI outperforms BMI, WC, and WHtR in predicting both cardiovascular and all-cause mortality risks (22, 23). Elevated WWI values have been linked to higher risks of hypertension, diabetes, coronary heart disease, and stroke (24–27). Recent research has further validated the use of WWI as the primary obesity metric, showing its superior ability to predict stroke risk, especially in older adults with hypertension. This capability allows for a more precise assessment compared to conventional metrics (28). Moreover, WWI exhibits a distinctive J-shaped association with cardiovascular disease risk, reflecting its ability to capture both low- and high-risk groups effectively, which is particularly pertinent for the studied population (29). These findings suggest that WWI offers a more robust and comprehensive metric for identifying obesity-related health risks, underscoring its utility in detecting individuals at elevated risk of adverse health outcomes, particularly among middle-aged and older Chinese adults.

Although the development of stroke involves a complex interplay of factors, obesity is recognized as a major contributor. However, the specific relationship between the WWI and stroke remains underexplored. In particular, there is limited evidence examining the connection between WWI and stroke risk within the Chinese population. This study seeks to fill this research gap by investigating the longitudinal association between WWI and stroke incidence in a Chinese cohort. With the prevalence of stroke steadily increasing among older adult individuals in China, our findings are expected to provide valuable insights that could support the development of targeted strategies for stroke prevention and management in this high-risk population.

## Materials and methods

### Study design

This study employed a prospective cohort design, utilizing data from the China Health and Retirement Longitudinal Study (CHARLS) spanning the years 2011–2020^1^ (30). The research targeted Chinese individuals aged 45 years and older, focusing on middle-aged and older adult populations. The WWI was examined as the primary independent variable. Stroke incidence, defined as a binary outcome (no stroke vs. stroke), was identified as the main dependent variable.

### Participants selection

Participants for this study were selected from the CHARLS, a nationally representative longitudinal cohort study organized by Peking University. CHARLS aims to generate high-quality data on the health, social, and economic conditions of middle-aged and older adults in China, offering valuable insights into aging-related topics such as chronic diseases, disability, and mortality. The dataset includes extensive information on demographics, health outcomes, physical functioning, and biomarkers, serving as a critical resource for public health and aging research. Initially launched in 2011, the investigation enrolled 17,708 participants across 28 provinces, spanning 150 counties/districts and 450 villages/urban neighborhoods. Subsequent follow-up surveys were conducted in 2013–2014 (wave 2), 2015–2016 (wave 3), 2017–2018 (wave 4), and 2019–2020 (wave 5), with blood samples collected during waves 1 and 3.

**Figure 1:**
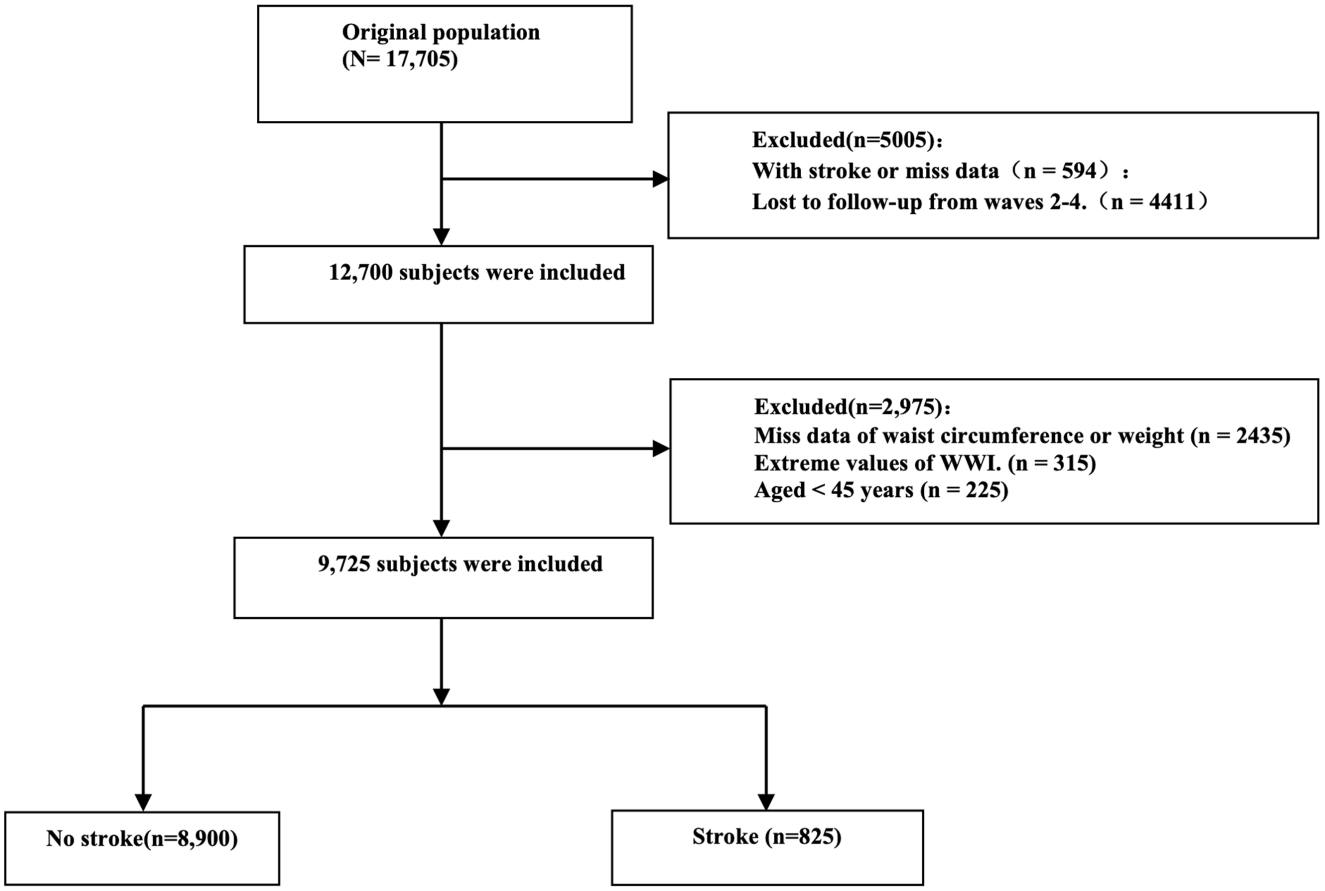
Flow chart visualizing the patient selection process.

This study analyzed participants from the 2011 baseline survey, using blood sample data collected during the initial wave. Follow-up was conducted through waves 2, 3, 4, and 5 to monitor stroke incidence. To ensure data reliability, individuals with extreme WC and weight values were excluded (31). Additionally, participants with a history of stroke at baseline or without follow-up data in any subsequent wave were removed from the analysis. The participant selection process is detailed in Figure 1.

### Variables

#### Clinical variables and laboratory variables

Demographic data, including age, sex, height(cm), weight(kg), marital status (married or other), BMI, smoking status (current, former, never), drinking status (current, former, never), education level (college and above, middle or high school, no formal education, primary school), and medical history (diabetes, hypertension, heart disease, dyslipidemia, kidney disease), were extracted from the CHARLS database. Laboratory assessments covered low-density lipoprotein cholesterol (LDL-C, mg/dL), high-density lipoprotein cholesterol (HDL-C, mg/dL), triglycerides (TG, mg/dL), total cholesterol (TC, mg/dL), creatinine (mg/dL), urea(mg/dL), glycated hemoglobin (HbA1c, %), and fasting glucose(mg/dL). The WWI was calculated by dividing waist circumference (cm) by the square root of weight (kg) (23).

#### Diagnosis of stroke

Participants without a history of stroke at baseline who reported experiencing a stroke during follow-up were classified as having a stroke event. Stroke events were identified through standardized questions: (i) “Were you told by your doctor that you had been diagnosed with a stroke?” (ii) “When were you first diagnosed/aware of the disease yourself?” (iii) “Are you currently receiving any follow-up treatment for your stroke?” Participants who provided positive responses during follow-up were categorized as having experienced a stroke. Those who reported a stroke in any of the four follow-up surveys were included in the stroke group, whereas individuals without stroke reports were placed in the no-stroke group. Participants who lacked follow-up data were excluded from the analysis.

### Statistical analysis

Continuous variables were described as mean ± standard deviation or median (interquartile range), with comparisons performed using the t-test or Mann–Whitney U test, depending on data distribution. Categorical variables were presented as percentages and analyzed using the chi-square test or Fisher’s exact test. Participant characteristics were evaluated across quartiles of the WWI.

To address missing data, participants with incomplete information for the independent variable (WWI) or the dependent variable (stroke incidence) were excluded, resulting in a final analytical sample of 9,725 out of 12,640 eligible individuals. In this prospective cohort study, missing data primarily involved the dependent variable (stroke incidence). For participants with complete WWI and stroke incidence data but missing one or more covariates, an available-case analysis approach was employed. This method leveraged all available data for each covariate, allowing individuals with incomplete data on other variables to remain in the analysis.

The relationship between WWI and stroke risk was analyzed using multivariate logistic regression. Logistic regression was selected due to the binary nature of the outcome variable (stroke) and the continuous nature of the primary predictor (WWI). To address potential confounding factors, a stepwise adjustment strategy was employed to develop the models. The Crude Model was unadjusted, Model 1 controlled for basic demographic variables (age and sex), Model 2 further included social and lifestyle factors (marital status, residence, education level, smoking, and drinking habits), and Model 3 accounted for clinical comorbidities (hypertension, dyslipidemia, diabetes, heart disease, and kidney disorders). This approach enabled a gradual evaluation of the influence of additional covariate groups on the WWI-stroke association while reducing the likelihood of overfitting. Effect sizes were expressed as odds ratios (OR) with 95% confidence intervals (95% CI). WWI was also categorized into quartiles, and trend tests were conducted to assess potential linear relationships between WWI and stroke. Effect sizes for these categorical analyses were similarly reported with 95% confidence intervals. Confounding variables were adjusted based on clinical knowledge and prior literature. To further explore the relationship between WWI and stroke incidence, restricted cubic spline analysis was performed. This method used 4 knots placed at the 5th, 35th, 65th, and 95th percentiles of WWI distribution, following established guidelines for optimal knot placement in epidemiological research. Model assumptions were systematically tested during the analysis. Linearity was evaluated by testing for non-linearity in the cubic spline models, and the robustness of the WWI-stroke association was verified through progressive adjustments. The study design ensured independence of observations, as each participant contributed only one observation to the analysis. Stratified analyses were conducted to examine the WWI-stroke association across subgroups defined by demographic and clinical characteristics, including age (<60 or ≥ 60 years), gender, residence (rural or urban), smoking status (current, former, or never), drinking status (current, former, or never), and the presence of comorbidities such as hypertension, dyslipidemia, diabetes, heart disease, and kidney disorders. Statistical significance was defined as *p* < 0.05. All analyses were performed using R statistical software (version 4.4.1).

## Results

### Baseline characteristics

The baseline characteristics of the 9,725 participants are presented in Table 1, grouped by WWI quartiles. Participants in the higher WWI quartiles were, on average, older, with mean age ranging from 55.53 ± 7.67 years in Q1 to 61.62 ± 8.97 years in Q4 (*p* < 0.0001). The proportion of females was markedly higher in Q4 (81.66%) compared to Q1 (32.12%) (*p* < 0.0001). The prevalence of stroke increased with higher WWI, from 6.03% in Q1 to 11.63% in Q4 (*p* < 0.0001). Furthermore, higher WWI quartiles were associated with higher BMI, waist circumference, and blood pressure levels, as well as higher prevalence of hypertension, dyslipidemia, diabetes, and heart problems (all *p* < 0.0001) (Table 1).

**Table 1:** Characteristics of participants.

Characteristics	Total	Q1 (<10.57)	Q2 (10.57–11.10)	Q3 (11.10–11.66)	Q4 (>11.66)	*p*
No.	9,725	2,420	2,427	2,421	2,467	
Age (years)	57.91 ± 8.57	55.53 ± 7.67	56.47 ± 8.02	57.94 ± 8.25	61.62 ± 8.97	<0.0001
Sex, (%)						<0.0001
Female	5,307 (54.60)	777 (32.12)	1,047 (43.32)	1,471 (60.81)	2012 (81.66)	
Male	4,412 (45.40)	1,642 (67.88)	1,370 (56.68)	948 (39.19)	452 (18.34)	
Residence						<0.0001
Rural	6,437 (66.19)	1710 (70.66)	1,569 (64.92)	1,546 (63.86)	1,612 (65.34)	
Urban	3,288 (33.81)	710 (29.34)	848 (35.08)	875 (36.14)	855 (34.66)	
Smoking, (%)						<0.0001
Current	2,922 (30.05)	1,098 (45.37)	850 (35.17)	620 (25.61)	354 (14.35)	
Former	740 (7.61)	200 (8.26)	236 (9.76)	170 (7.02)	134 (5.43)	
Never	6,063 (62.34)	1,122 (46.36)	1,331 (55.07)	1,631 (67.37)	1979 (80.22)	
Education level (%)						<0.0001
College and above	139 (1.43)	55 (2.27)	43 (1.78)	25 (1.03)	16 (0.65)	
Middle or high school	2,939 (30.23)	919 (37.98)	879 (36.38)	698 (28.84)	443 (17.96)	
Primary school	3,995 (41.09)	1,023 (42.27)	990 (40.98)	1,014 (41.90)	968 (39.24)	
No formal education	2,650 (27.25)	423 (17.48)	504 (20.86)	683 (28.22)	1,040 (42.16)	
Marital status, (%)						<0.0001
Married	8,707 (89.53)	2,214 (91.49)	2,208 (91.35)	2,190 (90.46)	2095 (84.92)	
Other	1,018 (10.47)	206 (8.51)	209 (8.65)	231 (9.54)	372 (15.08)	
Drinking, (%)						<0.0001
Current	1,328 (13.66)	380 (15.70)	386 (15.97)	296 (12.23)	266 (10.78)	
Former	2,581 (26.54)	870 (35.95)	757 (31.32)	594 (24.54)	360 (14.59)	
Never	5,816(59.80)	1,170 (48.35)	1,274 (52.71)	1,531 (63.24)	1841 (74.63)	
WC (cm)	85.07 ± 9.83	76.61 ± 6.73	83.64 ± 7.60	87.59 ± 8.36	92.31 ± 8.99	<0.0001
Weight (Kg)	58.75 ± 10.66	57.66 ± 9.06	60.01 ± 10.65	59.97 ± 11.32	57.41 ± 11.15	<0.0001
BMI (kg/m2)	23.46 ± 3.55	21.78 ± 2.76	23.29 ± 3.12	24.16 ± 3.55	24.59 ± 3.99	<0.0001
Glucose (mg/dL)	109.22 ± 33.74	103.79 ± 26.95	109.25 ± 34.87	110.68 ± 31.53	112.83 ± 39.31	<0.0001
HbA1c, %	5.25 ± 0.75	5.12 ± 0.58	5.21 ± 0.78	5.27 ± 0.71	5.38 ± 0.89	<0.0001
Creatinine (mg/dL)	0.77 ± 0.18	0.80 ± 0.17	0.79 ± 0.19	0.76 ± 0.18	0.73 ± 0.17	<0.0001
Urea (mg/dL)	4.39 ± 1.22	4.39 ± 1.17	4.49 ± 1.26	4.41 ± 1.25	4.29 ± 1.18	<0.0001
TC (mg/dL)	194.25 ± 38.51	186.38 ± 36.65	191.79 ± 37.34	197.70 ± 39.23	200.57 ± 39.14	<0.0001
HDL cholesterol (mg/dL)	51.33 ± 15.21	54.61 ± 15.57	51.26 ± 15.14	49.84 ± 15.11	49.77 ± 14.54	<0.0001
LDL cholesterol (mg/dL)	117.04 ± 34.70	111.81 ± 32.34	115.27 ± 32.91	119.53 ± 35.99	121.22 ± 36.45	<0.0001
TG (mg/dL)	133.22 ± 110.93	108.84 ± 85.48	130.08 ± 108.11	144.29 ± 125.65	148.25 ± 115.34	<0.0001
SBP (mmHg)	129.22 ± 20.77	124.31 ± 18.35	128.05 ± 20.01	129.71 ± 20.71	134.70 ± 22.42	<0.0001
DBP (mmHg)	75.61 ± 11.88	73.92 ± 11.64	75.62 ± 12.08	76.10 ± 11.92	76.77 ± 11.71	<0.0001
Stroke, (%)	825 (8.48)	146 (6.03)	189 (7.82)	203 (8.38)	287 (11.63)	<0.0001
Hypertension, (%)	2072 (21.42)	299 (12.43)	464 (19.30)	587 (24.40)	722 (29.36)	<0.0001
Dyslipidemia, (%)	788 (8.26)	127 (5.35)	182 (7.70)	230 (9.69)	249 (10.26)	<0.0001
Diabetes, (%)	464 (4.81)	51 (2.12)	109 (4.54)	135 (5.64)	169 (6.91)	<0.0001
Heart Problems, (%)	971 (10.03)	181 (7.52)	211 (8.77)	257 (10.67)	322 (13.09)	<0.0001
Kidney disease, (%)	579 (5.99)	168 (6.99)	132 (5.49)	143 (5.94)	136 (5.53)	0.10

Continuous data are shown as mean ± SD (normal distribution) or median (quartile) (skewed distribution). Categorical data are shown as *n* (%).

WWI, Weight-Adjusted Waist Index; BMI, Body Mass Index; WC, Waist Circumference; LDL cholesterol, Low-Density Lipoprotein Cholesterol; HDL cholesterol, High-Density Lipoprotein Cholesterol; TG, Triglycerides; TC, Total Cholesterol; HbA1c, Glycated Hemoglobin; SBP, Systolic Blood Pressure; DBP, Diastolic Blood Pressure.

Q1, Weight-Adjusted Waist Index < 10.57; Q2, 10.57 ≤ Weight-Adjusted Waist Index < 11.10; Q3, 11.10 ≤ Weight-Adjusted Waist Index < 11.66; Q4, 11.66 ≤ Weight-Adjusted Waist Index.

### The relationship between the WWI and stroke

**Figure 2:**
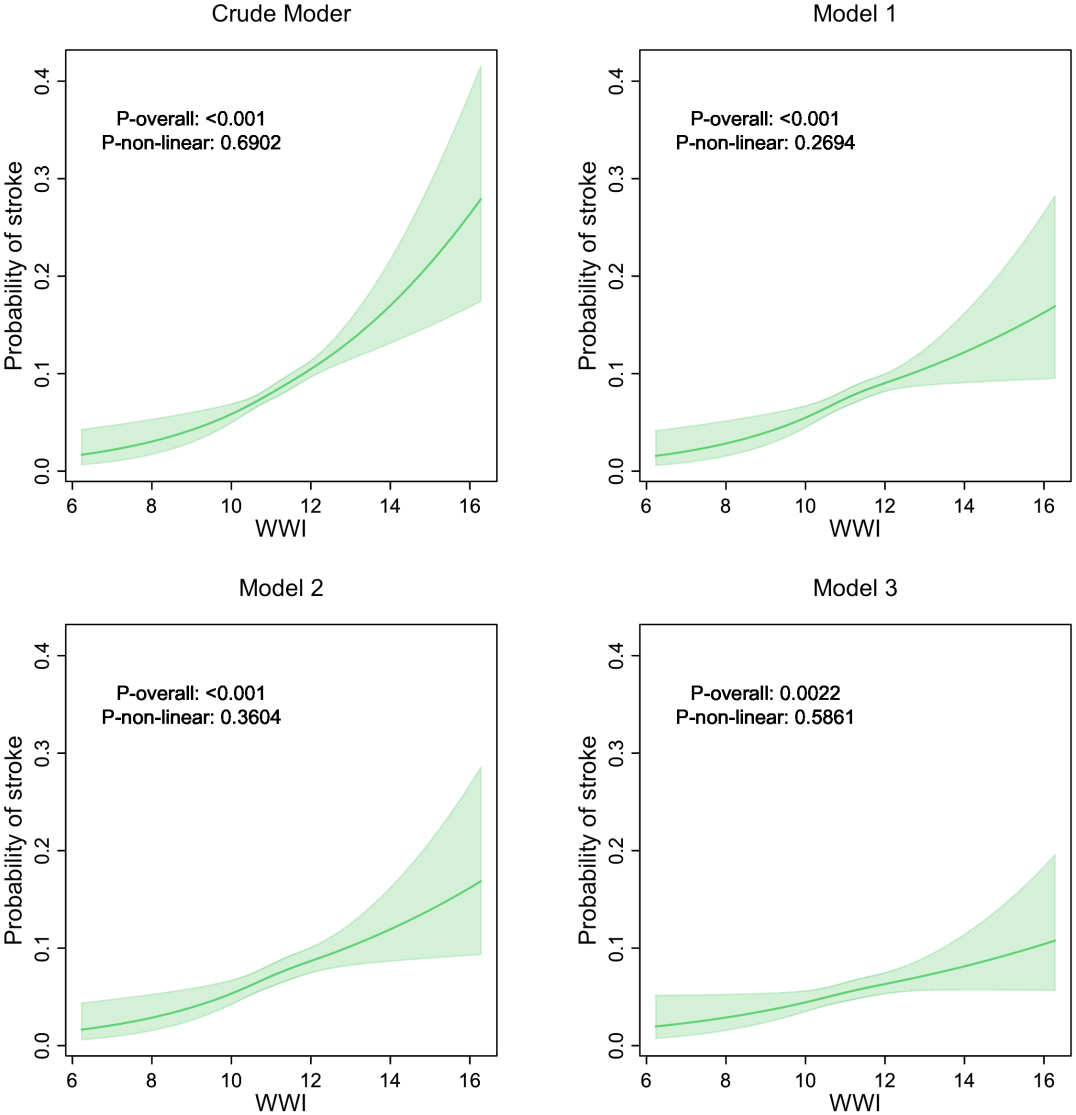
The relationship between WWI and the incidence of stroke.

The relationship between the WWI and stroke incidence was evaluated through multivariate logistic regression analyses and curve fitting. Results from Table 2 demonstrate that elevated WWI is consistently linked to an increased risk of stroke across all models. In the crude model, the OR for stroke was 1.35 (95% CI: 1.25, 1.46), which remained significant after adjustments in Model 1 (OR: 1.26, 95% CI: 1.15, 1.38), Model 2 (OR: 1.26, 95% CI: 1.15, 1.38), and Model 3 (OR: 1.18, 95% CI: 1.08, 1.30). Trend analyses confirmed a strong linear relationship between WWI and stroke risk (*p* for trend <0.0001 in the crude model and Model 1, and 0.001 in Model 3). Figure 2 illustrates the probability of stroke across different levels of WWI using restricted cubic spline curve fitting. The fitted curves show the relationship between WWI and predicted stroke probability for each model. The overall association between WWI and stroke risk was significant in all models (*p* < 0.001 for crude model, Model 1, and Model 2; *p* = 0.0022 for Model 3). The *p*-values for non-linearity were 0.6902, 0.2694, 0.3604, and 0.5861 for crude model, Model 1, Model 2, and Model 3, respectively. The curves demonstrate a generally positive association between WWI and predicted stroke probability across the range of observed WWI values in all models.

**Table 2:** Multivariate logistic regression analyses of WWI and stroke.

	Crude model		Model 1		Model 2		Model 3	
Variable	OR (95%CI)	*p*	OR (95%CI)	*p*	OR (95%CI)	*p*	OR (95%CI)	*p*
WWI	1.35 (1.25, 1.46)	<0.0001	1.26 (1.15, 1.38)	<0.0001	1.26 (1.15, 1.38)	<0.0001	1.18 (1.08, 1.30)	<0.001
WWI quartile								
Q1 (< 10.57)	Ref		Ref		Ref		Ref	
Q2 (10.57–11.09)	1.32 (1.06, 1.65)	0.01	1.3 (1.04, 1.62)	0.02	1.28 (1.02, 1.60)	0.03	1.21 (0.96, 1.52)	0.11
Q3 (11.10–11.66)	1.43 (1.14, 1.78)	0.002	1.36 (1.09, 1.71)	0.01	1.34 (1.07, 1.69)	0.01	1.19 (0.94, 1.51)	0.14
Q4 (> 11.66)	2.05 (1.67, 2.52)	<0.0001	1.8 (1.42, 2.27)	<0.0001	1.77 (1.40, 2.23)	<0.0001	1.52 (1.19, 1.92)	<0.001
*p* for trend		<0.0001		<0.0001		<0.0001		0.001

Crude model 1: no variables are adjusted.

Model 1 adjust for: sex and age.

Model 2 adjust for: sex, age, Marital status, Residence, education, Smoking, Drinking.

Model 3 adjust for: sex, age, Marital status, Residence, Education level, Smoking, Drinking, Hypertension, Dyslipidemia, Diabetes, Heart Problems, Kidney disease.

### Strata analyses

**Figure 3:**
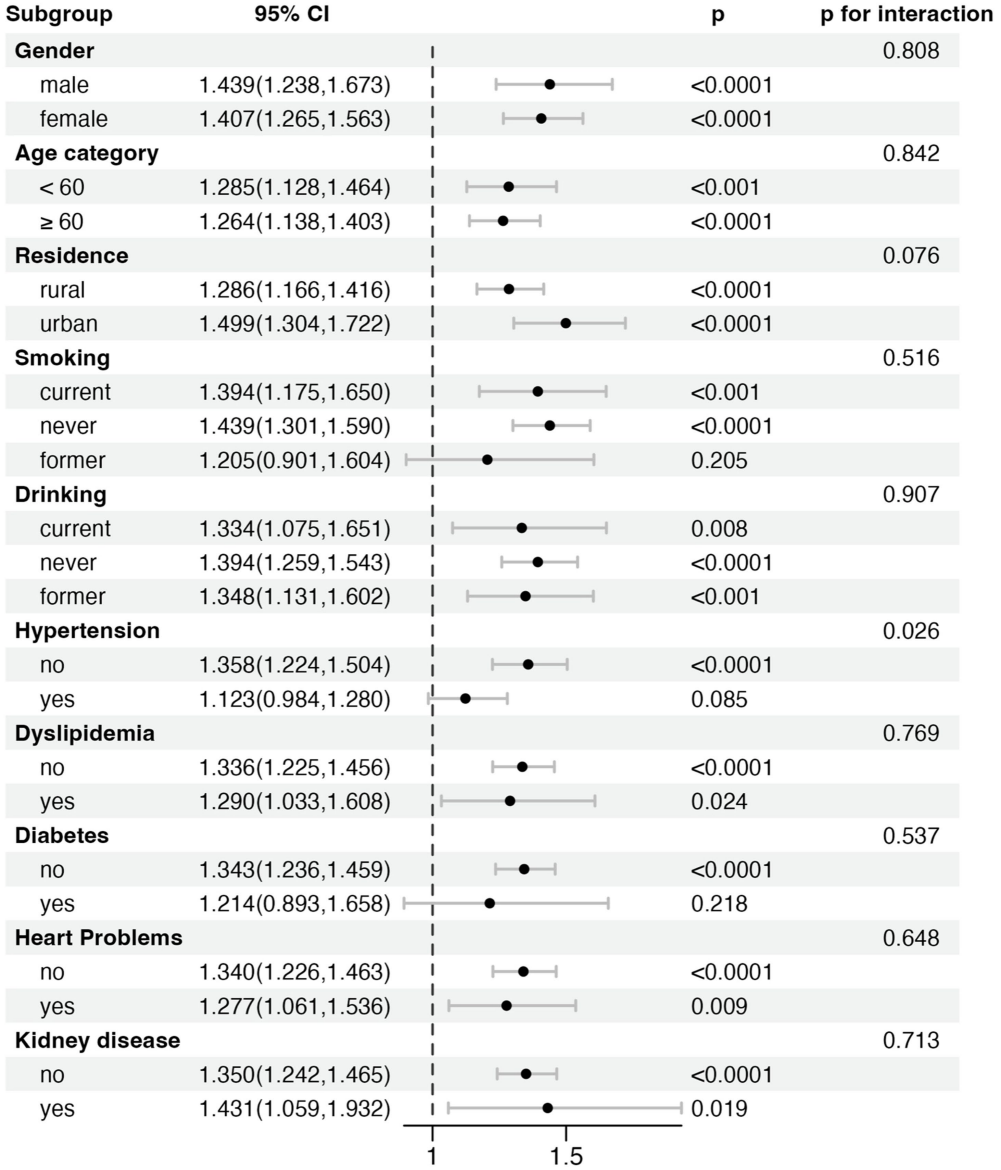
Strata analysis of the association between Weight-Adjusted Waist Index (WWI) and stroke risk across different subgroups.

Strata analyses were conducted to evaluate the consistency of the relationship between the WWI and stroke across different demographic and clinical subgroups (Figure 3). These subgroups included sex, age, residence, smoking status, drinking status, hypertension, dyslipidemia, diabetes, heart problems, and kidney disease. The findings demonstrated that the positive correlation between higher WWI and increased stroke risk was generally consistent across all subgroups. Significant interactions were observed in the strata based on hypertension (*p* for interaction = 0.026), suggesting that the association between WWI and stroke might be stronger in participants without hypertension. In contrast, no notable interactions were observed in other strata, indicating the robustness of the WWI-stroke relationship across different population segments.

### Sensitivity analysis

To investigate the potential impact of loss to follow-up on our findings, we compared baseline characteristics between participants included in the analysis (*n* = 9,725) and those lost to follow-up (*n* = 2,915) (Table 3). This analysis revealed significant differences in numerous baseline characteristics, including age, gender, residence, education level, smoking and drinking status, and various clinical variables. In terms of clinical variables, the excluded group showed significantly higher levels of prevalent hypertension, diabetes, heart problems and kidney disease. Notably, the WWI was significantly higher in the excluded group (11.15 ± 0.86 vs. 11.33 ± 0.98, *p* < 0.0001). In addition, we performed two further sensitivity analyses to assess the robustness of our findings. First, we excluded participants with extreme WWI values identified using the Boxplot method, and the results remained consistent (Supplementary Table S1). Second, we reanalyzed the data using alternative WWI categorization methods (tertiles instead of quartiles), and this also demonstrated similar associations (Supplementary Table S2). These analyses reinforce the robustness of our findings under different analytical assumptions.

**Table 3:** Baseline characteristics of participants included in the analysis compared to those excluded due to loss to follow-up.

Characteristics	Total (*n* = 12640)	Include (*n* = 9725)	Exclude (*n* = 2915)	*P*
Age (years)	59.34 ± 9.57	57.91 ± 8.57	64.12 ± 11.09	<0.0001
Sex, (%)				<0.0001
Female	6649(52.64)	5307(54.60)	1342(46.07)	
Male	5983(47.36)	4412(45.40)	1571(53.93)	
Residence				<0.0001
Rural	8024(63.48)	6437(66.19)	1587(54.44)	
Urban	4616(36.52)	3288(33.81)	1328(45.56)	
Smoking, (%)				<0.0001
Current	3898(30.85)	2922(30.05)	976(33.52)	
Former	1126(8.91)	740(7.61)	386(13.26)	
Never	7613(60.24)	6063(62.34)	1550(53.23)	
Education level (%)				<0.0001
College and above	214(1.69)	139(1.43)	75(2.58)	
Middle or high school	3678(29.11)	2939(30.23)	739(25.39)	
Primary school	3582(28.35)	2650(27.25)	932(32.02)	
No formal education	5160(40.84)	3995(41.09)	1165(40.02)	
Marital status, (%)				<0.0001
Married	10985(86.91)	8707(89.53)	2278(78.15)	
Other	1655(13.09)	1018(10.47)	637(21.85)	
Drinking, (%)				<0.01
Current	1793(14.19)	1328(13.66)	465(15.96)	
Former	3368(26.65)	2581(26.54)	787(27.02)	
Never	7477(59.16)	5816(59.80)	1661(57.02)	
WC (cm)	85.12 ± 9.96	85.07 ± 9.83	85.29 ± 10.38	0.31
Weight (Kg)	58.44 ± 10.91	58.75 ± 10.66	57.42 ± 11.64	<0.0001
BMI (kg/m2)	23.33 ± 3.60	23.46 ± 3.55	22.89 ± 3.71	<0.0001
WWI	11.19 ± 0.90	11.15 ± 0.86	11.33 ± 0.98	<0.0001
Glucose (mg/dL)	110.38 ± 37.62	109.22 ± 33.74	114.89 ± 49.64	<0.0001
HbA1c, %	5.27 ± 0.82	5.25 ± 0.75	5.34 ± 1.02	<0.001
Creatinine (mg/dL)	0.78 ± 0.24	0.77 ± 0.18	0.83 ± 0.38	<0.0001
Urea (mg/dL)	4.45 ± 1.25	4.39 ± 1.22	4.68 ± 1.37	<0.0001
TC cholesterol (mg/dL)	193.80 ± 38.92	194.25 ± 38.51	192.07 ± 40.44	0.04
HDL cholesterol (mg/dL)	51.32 ± 15.35	51.33 ± 15.21	51.28 ± 15.89	0.91
LDL (mg/dL)	116.63 ± 35.07	117.04 ± 34.70	115.05 ± 36.42	0.03
TG (mg/dL)	132.90 ± 108.55	133.22 ± 110.93	131.66 ± 98.78	0.55
SBP (mmHg)	130.67 ± 21.55	129.22 ± 20.77	135.58 ± 23.35	<0.0001
DBP (mmHg)	75.89 ± 12.14	75.61 ± 11.88	76.84 ± 12.93	<0.0001
Hypertension, (%)	2935(23.34)	2072(21.42)	863(29.73)	<0.0001
Dyslipidemia, (%)	1056(8.52)	788(8.26)	268(9.39)	0.06
Diabetes, (%)	697(5.56)	464(4.81)	233(8.07)	<0.0001
Heart Problems, (%)	1452(11.54)	971(10.03)	481(16.57)	<0.0001
Kidney disease, (%)	797(6.34)	579(5.99)	218(7.52)	<0.01

Notes: Continuous data are shown as mean ± SD (normal distribution) or median (quartile) (skewed distribution). Categorical data are shown as n (%).

Abbreviations: WWI: Weight-Adjusted Waist Index; BMI: Body Mass Index; WC: Waist Circumference; LDL cholesterol: Low-Density Lipoprotein Cholesterol; HDL cholesterol: High-Density Lipoprotein Cholesterol; TG: Triglycerides; TC: Total Cholesterol; HbA1c: Glycated Hemoglobin; SBP: Systolic Blood Pressure; DBP: Diastolic Blood Pressure;

## Discussion

This study investigated the relationship between WWI and stroke incidence in aged 45 and older Chinese population. The results identified a significant association between elevated WWI levels and an increased risk of stroke. This relationship remained consistent across various models, even after adjusting for demographic, lifestyle, and health-related factors. Multivariate logistic regression analyses showed that individuals in the highest WWI quartile had a substantially higher stroke risk compared to those in the lowest quartile. Stratified analyses and sensitivity analysis further validated the robustness of this association across different demographic and clinical subgroups, with notable interactions observed among participants with hypertension. These findings highlight the potential of WWI as an effective predictor of stroke risk in this population, supporting the study’s objective of evaluating WWI’s utility in forecasting adverse health outcomes.

The WWI, a novel obesity metric, adjusts WC by body weight to better represent the distribution of fat and muscle mass (21). This unique characteristic enhances WWI’s ability to predict health risks associated with obesity. Elevated WWI levels are indicative of excessive abdominal fat accumulation (32), which is strongly linked to key stroke risk factors, including metabolic disturbances, vascular dysfunction, and inflammatory responses (33, 34). Dysfunctional adipose tissue contributes to the release of pro-inflammatory cytokines, such as interleukins and tumor necrosis factors, which play critical roles in the development, progression, and rupture of atherosclerotic plaques, ultimately leading to thromboembolic events (35–37). Central obesity also promotes oxidative stress, which can cause endothelial dysfunction and microvascular remodeling, potential mechanisms underlying stroke (33, 38). Compared with traditional metrics like BMI and WC, WWI provides a more accurate assessment of central obesity, which is crucial for evaluating stroke risk. Research has demonstrated that WWI is a superior predictor of cardiovascular disease and all-cause mortality compared to BMI and WC (27, 39). This makes WWI a more comprehensive metric for assessing individual health and predicting stroke risk, particularly in aged 45 and older populations. Moreover, higher WWI levels may increase stroke risk through the secretion of adipokines such as leptin and adiponectin, which are involved in pro-inflammatory states, oxidative stress, and endothelial dysfunction. Elevated leptin levels have been associated with higher stroke risk, while reduced adiponectin levels are linked to vascular dysfunction and microvascular damage (40, 41). Additionally, central obesity correlates with increased inflammatory markers, such as C-reactive protein and interleukin-6, which contribute to atherosclerotic plaque instability and vascular remodeling (42). These mechanisms may be further influenced by lifestyle and environmental factors unique to the Chinese population. High salt intake, a common dietary habit, may exacerbate the proinflammatory and vascular effects of visceral adiposity, while reduced physical activity levels, particularly among urban-dwelling adults, could enhance the adverse metabolic effects of central obesity (43–45). By more effectively capturing fat distribution disparities compared to BMI or WC, WWI offers distinct advantages in predicting obesity-related stroke risk.

Our study demonstrates a significant association between elevated WWI and an increased risk of stroke in aged 45 and older Chinese adults. These findings align with the results of Jiayi et al. (24), which also identified WWI as a strong predictor of stroke risk. However, our research uniquely focuses on Chinese individuals aged 45 and above, utilizing prospective cohort data collected from 2011 to 2020. This approach offers a higher level of evidence compared to Jiayi et al.’s (24) cross-sectional analysis, which supports the WWI-stroke relationship but provides comparatively weaker evidence. Previous studies have examined the relationship between WWI and various health outcomes, including all-cause mortality, cardiovascular disease, and hypertension, though their focus and populations differ. For instance, Shuang Cai et al. (22) investigated the association between WWI and all-cause mortality in an older adult Chinese cohort, reporting that higher WWI correlates with increased mortality risk. While their research and ours both emphasize older adult Chinese populations, our study specifically addresses stroke risk, providing a more targeted understanding of WWI’s predictive utility. Similarly, Jiao Wang et al. (46) explored the relationship between WWI and hypertension in an older adult American population (aged 60 and above), finding that higher WWI is significantly associated with hypertension risk. Despite focusing on different health outcomes, both studies underline WWI’s effectiveness as a predictive tool in older populations. Further studies by Zhang et al. and Li et al. (27, 39) analyzed the relationship between WWI and risks of all-cause mortality, cardiovascular mortality, and specific cardiovascular disease subtypes in adult populations from China and the United States. While these studies reinforce the validity of WWI as a predictor of disease risk, our research is distinct in its prospective validation of the direct association between WWI and stroke risk in a middle-aged and older Chinese population. This focus provides novel insights that hold significant public health implications. Additionally, our study benefits from a longer follow-up period and more comprehensive statistical analyses, enhancing the causal inference of the WWI-stroke association. These methodological strengths further establish the robustness of our findings, highlighting WWI’s utility as a valuable metric for predicting stroke risk in this population.

Our stratified analysis identified a significant interaction between WWI and hypertension status (*p* for interaction = 0.026), suggesting that the association between WWI and stroke risk differs by hypertension status. Specifically, WWI was strongly associated with stroke risk in non-hypertensive individuals, whereas this relationship was weaker and not statistically significant in hypertensive individuals. This may reflect the dominant role of hypertension as a stroke risk factor, potentially attenuating the additional impact of visceral fat. In contrast, in non-hypertensive individuals, visceral fat may play a more direct role in increasing stroke risk through mechanisms such as insulin resistance, chronic inflammation, and endothelial dysfunction. Additionally, the potential risk of overfitting was carefully addressed in this study. With a sample size of 9,725 participants and 825 stroke events (8.48%), allowing for the inclusion of 12 covariates while maintaining sufficient statistical power. Sensitivity analyses and stratified analyses reinforced the robustness of these findings, suggesting that the observed associations are not likely to result from overfitting. Future research should consider employing formal cross-validation techniques or using information criteria to further validate these results, ensuring the reliability and generalizability of the findings.

This study has several limitations. First, stroke events were self-reported, which may have introduced recall bias or misclassification, particularly underreporting among individuals with mild or asymptomatic strokes. Such non-random misclassification may have resulted in an underestimation of the true association between WWI and stroke risk. Nonetheless, prior studies have shown that self-reported stroke diagnoses generally exhibit high validity (47, 48), supporting the reliability of our findings. Second, the study did not fully account for potential confounding factors such as diet, physical activity, and genetic predisposition, which may have influenced the observed associations. Third, a significant limitation was the loss to follow-up of 2,915 participants (23.1%). As outlined in Table 3, individuals lost to follow-up were older, exhibited a higher prevalence of cardiovascular risk factors, and had a higher mean WWI at baseline. This non-random attrition likely led to an underestimation of the true association between WWI and stroke risk, as participants in poorer health at baseline, and therefore at greater risk of stroke, were more likely to be excluded from the final analysis. Finally, baseline WWI measurements were used without accounting for potential changes over time, which may have introduced some imprecision into the risk estimates. This study demonstrates a statistical association between baseline WWI and stroke risk but does not establish causality. Future research involving diverse populations and enhanced follow-up strategies is needed to validate and expand upon these findings.

## Conclusion

This prospective cohort study identified a significant association between elevated WWI and an increased risk of stroke in aged 45 and older Chinese adults. The results emphasize WWI as a reliable predictor of stroke, with the relationship consistently observed across various demographic and clinical subgroups. Future research should focus on validating these findings in diverse populations and exploring the biological mechanisms underlying the WWI-stroke relationship, particularly pathways related to inflammation and vascular dysfunction. Integrating WWI into existing risk prediction tools has the potential to enhance strategies for stroke prevention and management.

## Data Availability

Publicly available datasets were analyzed in this study. This data can be found here: https://charls.pku.edu.cn/.

## Footnotes

^1^ https://charls.pku.edu.cn/
