# Supplementary material for: Longitudinal relationship between baseline Weight-Adjusted Waist Index and stroke risk over 8 years in Chinese adults aged 45 and older: a prospective cohort study
Source: Front Public Health. 2025 Feb 12;13:1505364. doi: 10.3389/fpubh.2025.1505364 (PMC11861358; doi:10.3389/fpubh.2025.1505364)
Supplement: Supplementary file 1 [file Table_1.docx]

Supplementary Table S1: Multivariate logistic regression analyses of WWI and stroke.

|  | Crude model |  | Model 1 |  | Model 2 |  | Model 3 |  |
| --- | --- | --- | --- | --- | --- | --- | --- | --- |
| Variable | OR (95%CI) | *P* | OR (95%CI) | *P* | OR (95%CI) | *P* | OR (95%CI) | *P* |
| WWI | 1.30(1.27,1.53) | <0.0001 | 1.30(1.17,1.45) | <0.0001 | 1.31(1.18,1.45) | <0.0001 | 1.20(1.08,1.36) | <0.001 |

Notes:

Crude model 1: no variables are adjusted.

Model 1 adjust for: sex and age.

Model 2 adjust for: sex, age, Marital status, Residence, education, Smoking, Drinking

Model 3 adjust for: sex, age, Marital status, Residence, Education level, Smoking, Drinking, Hypertension, Dyslipidemia, Diabetes, Heart Problems, Kidney disease.
