# Supplementary material for: Longitudinal relationship between baseline Weight-Adjusted Waist Index and stroke risk over 8 years in Chinese adults aged 45 and older: a prospective cohort study
Source: Front Public Health. 2025 Feb 12;13:1505364. doi: 10.3389/fpubh.2025.1505364 (PMC11861358; doi:10.3389/fpubh.2025.1505364)
Supplement: Supplementary file 2 [file Table_2.docx]

Supplementary Table S2: Multivariate logistic regression analyses of WWI and stroke.

|  | Crude model |  | Model 1 |  | Model 2 |  | Model 3 |  |
| --- | --- | --- | --- | --- | --- | --- | --- | --- |
| Variable | OR (95%CI) | *P* | OR (95%CI) | *P* | OR (95%CI) | *P* | OR (95%CI) | *P* |
| WWI Tertile |  |  |  |  |  |  |  |  |
| Q1( < 10.57) | Ref |  | Ref |  | Ref |  | Ref |  |
| Q2(10.57 - 11.09) | 1.25(1.03,1.50) | 0.02 | 1.21(0.97,1.46) | 0.05 | 1.20(0.99,1.46) | 0.06 | 1.10(0.90,1.34) | 0.33 |
| Q3(11.10-11.66) | 1.86(1.56,2.22) | 0.00 | 1.67(1.37,2.04) | 0.00 | 1.68(1.38,2.04) | 0.00 | 1.44(1.18,1.76) | 0.00 |
| *p* for trend |  | <0.001 |  | <0.001 |  | <0.001 |  | <0.001 |

Notes:

Crude model 1: no variables are adjusted.

Model 1 adjust for: sex and age.

Model 2 adjust for: sex, age, Marital status, Residence, education, Smoking, Drinking

Model 3 adjust for: sex, age, Marital status, Residence, Education level, Smoking, Drinking, Hypertension, Dyslipidemia, Diabetes, Heart Problems, Kidney disease.
